# Supplementary material for: Scoping Review on Platelets and Tumor Angiogenesis: Do We Need More Evidence or Better Analysis?
Source: Int J Mol Sci. 2022 Nov 2;23(21):13401. doi: 10.3390/ijms232113401 (PMC9656254; doi:10.3390/ijms232113401)
Supplement: Supplementary file 1 [file ijms-23-13401-s001.zip › ijms-1953395-supplementary.pdf]

Table S1. Search strategy

PUBMED
(platelet[title] or platelet[mesh]) AND (tumor-progression[title/abstract] OR cancer-progression[title/abstract] OR ((angiogenesis[title/abstract] OR “drug resistance”[title/abstract] OR “therapeutic effect”[title/abstract] OR “therapeutic effects”[title/abstract]) AND (tumor[title/abstract] or cancer[title/abstract] or cancer[mesh] OR cancers[title/abstract] OR tumors[title/abstract]))) NOT ("case report"[title] OR "case series"[title])
ISI OF KNOWLEDGE
